# Performance of an affordable urine self-sampling method for human papillomavirus detection in Mexican women

**DOI:** 10.1371/journal.pone.0254946

**Published:** 2021-07-21

**Authors:** Rubí Hernández-López, Luis Hermosillo, Leith León-Maldonado, Rafael Velázquez-Cruz, Leticia Torres-Ibarra, Eduardo Lazcano-Ponce, Attila Lörincz, Cosette M. Wheeler, F. Xavier Bosch, Jack Cuzick, Berenice Rivera-Paredez, Belinda Nedjai, Jorge Salmerón

**Affiliations:** 1 Oficina de Análisis del Plan de Salud, Subgerencia Técnica del Plan de Salud, Gerencia de Administración del Plan de Salud, Banco de México, Mexico City, Mexico; 2 Facultad de Medicina, Centro de Investigación en Políticas, Población y Salud, Universidad Nacional Autónoma de México, Mexico City, Mexico; 3 Consejo Nacional de Ciencia y Tecnología-Centro de Investigación en Salud Poblacional, Instituto Nacional de Salud Pública, Cuernavaca, Morelos, Mexico; 4 Laboratorio de Genómica del Metabolismo Óseo, Instituto Nacional de Medicina Genómica (INMEGEN), Mexico City, Mexico; 5 Centro de Investigación en Salud Poblacional, Instituto Nacional de Salud Pública, Cuernavaca, Morelos, Mexico; 6 Centre for Cancer Prevention, Wolfson Institute of Preventive Medicine, Queen Mary University of London, London, United Kingdom; 7 Department of Pathology and Obstetrics & Gynecology, University of New Mexico Comprehensive Cancer Center, Albuquerque, New Mexico, United States of America; 8 Unit of Infections and Cancer—Information and Interventions, Cancer Epidemiology Research Programme, Catalan Institute of Oncology (ICO)—IDIBELL, l’Hospitalet de Llobregat, Open University of Catalonia, Barcelona, Spain; 9 Facultad de Medicina, Centro de Investigación en Políticas, Población y Salud, Universidad Nacional Autónoma de México, Mexico City, Mexico; Istituto Nazionale Tumori IRCCS Fondazione Pascale, ITALY

## Abstract

**Introduction:**

Urine self-sampling for human papillomavirus (HPV)-based cervical cancer screening is a non-invasive method that offers several logistical advantages and high acceptability, reducing barriers related to low screening coverage. This study developed and evaluated the performance of a low-cost urine self-sampling method for HPV-testing and explored the acceptability and feasibility of potential implementation of this alternative in routine screening.

**Methods:**

A series of sequential laboratory assays examined the impact of several pre-analytical conditions for obtaining DNA from urine and subsequent HPV detection. Initially, we assessed the effect of ethylaminediaminetetraacetic acid (EDTA) as a DNA preservative examining several variables including EDTA concentration, specimen storage temperature, time between urine collection and DNA extraction, and first-morning micturition versus convenience sample collection. We further evaluated the agreement of HPV-testing between urine and clinician-collected cervical samples among 95 women. Finally, we explored the costs of self-sampling supplies as well as the acceptability and feasibility of urine self-sampling among women and healthcare workers.

**Results:**

Our results revealed higher DNA concentrations were obtained when using a 40mM EDTA solution, storing specimens at 25°C and extracting DNA within 72 hrs. of urine collection, regardless of using first-morning micturition or a convenience sampling. We observed good agreement (Kappa = 0.72) between urine and clinician-collected cervical samples for HPV detection. Furthermore, urine self-sampling was an affordable method (USD 1.10), well accepted among cervical cancer screening users, healthcare workers, and decision-makers.

**Conclusion:**

These results suggest urine self-sampling is feasible and appropriate alternative for HPV-testing in HPV-based screening programs in lower-resource contexts.

## Introduction

Cervical cancer is the second leading cause of cancer deaths in low- and middle-income countries (LMICs), with almost 285,000 new cases every year [1]. Current screening programs, in these settings, have low coverage, being 59.7% in Mexico, and even lower in other countries [2,3]. Cervical screening requires trained personnel and infrastructures to collect cervical samples, which is not always available in LMICs [4–6]. Furthermore, cultural background alone can act as a barrier to achieving high screening coverage when patients’ acceptance of pelvic examination is low [4,5].

Urine self-sampling has been proposed as an alternative primary screening method for detecting high-risk human papillomavirus (hrHPV), given self-sampling may increase women’s willingness to participate in screening [3–5]. Previous studies have used specially designed collection devices with specialized buffers and preservation media, which are costly [7,8], hindering implementation of urine sampling for hrHPV screening in LMICs, where it is most needed [1,6].

There are several challenges to obtaining proper urine DNA preservation in order to achieve an easy and affordable method applicable to real-world conditions. Among the main barriers are: 1) time between sampling and processing, as studies have shown that urine DNA nucleases reduce concentrations of DNA in urine over time [5,9]; 2) an optimal concentration of ethylenediaminetetraacetic acid (EDTA) is needed for reducing DNA nucleases activity [5,7,10]; 3) sample storage temperature has also been shown to affect urine DNA preservation [9,10]; and 4) it is unclear whether using first-morning micturition is superior to a convenience sample from any time of day [11,12].

It is key to have simple and low-cost urine collection and preservation procedures able to overcome previously described difficulties that may allow use of self-collected urine samples for the detection of hrHPV as a primary test for cervical cancer screening [13,14]. hrHPV-testing in urine samples is a non-invasive method that may provide enormous logistical advantages and higher acceptance as compared to samples collected by clinicians during a pelvic examination. The possibility of using urine for hrHPV-based cervical cancer screening could overcome barriers that limit screening coverage [4,5,15]. It is also important to evaluate the acceptability of urine self-collection among participants of the targeted cervical cancer screening program, among health professionals involved in the program and decision-makers of healthcare-services who are responsible for potential implementation of this procedure within the cervical cancer screening program.

We aimed to evaluate a low-cost method for urine self-sampling for HPV-testing that could be potentially translated into clinical practice and public policy, requiring minimum training among healthcare providers. Additionally, cost, acceptability and feasibility were evaluated as core elements for implementation.

## Materials and methods

### Study design

The study was conducted in two phases. During the first phase, we conducted a series of sequential laboratory evaluations to examine the impact of different pre-analytical conditions on the DNA concentration of stored urine samples. All these evaluations were performed in a set of urine samples donated by five volunteer women, aged 25 to 45, postgraduate students from Mexico City. The second phase was conducted to evaluate the agreement between hrHPV-testing in urine samples and clinician-collected cervical samples. Finally, we explored the acceptability, perception of feasibility and costs of urine self-sampling for hrHPV-testing in cervical cancer screening. The IRB of the National Public Health Institute (INSP) (CI:1417 CB: 1408–2016) approved the first phase protocol. The second phase was conducted within the FASTER study, reviewed and approved by the IRB of the INSP (Num: 1322–2015 and 1417–2016), the Mexican regulatory agency Federal Committee for Protection from Sanitary Risks (COFEPRIS) (163300410A0160/2016), and the Mexico City Ministry of Health (211/001/003/16); and registered in ClinicalTrials.gov, number: NCT03105856. The objective of the study, procedures, and possible consequences were explained to potential participants before obtaining written informed consent [16].

### First phase

**Fig 1:**
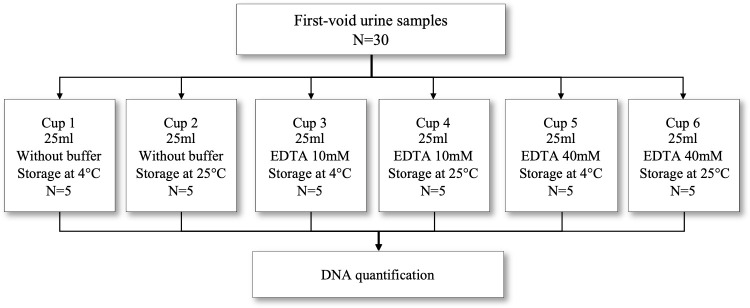
Overview of collection and management of urine samples to evaluate optimal EDTA concentration and storage temperature. This evaluation was performed in a set of urine samples from five volunteer women, who collected a first-morning, first-void urine sample, in six different cups (25ml each). Cups contained different EDTA concentrations added prior to urine addition: No EDTA, 10mM and 40mM EDTA. Half of the samples (the ones labeled with odd numbers) were stored at room temperature and those with even numbers were stored in the fridge at ~4°C. DNA extraction and quantification were performed 24 hours after collection.

We first evaluated the impact of different EDTA concentrations, and different storage temperatures on DNA concentration in urine samples (Fig 1). Five volunteer women were asked to collect a first-morning, first-void urine sample of ~25ml in each of six different cups, numbered from one to six. Cups contained different EDTA concentrations: no EDTA, 10mM and 40mM EDTA. Participants were instructed to store odd-numbered cups at room temperature and to store even numbered cups in their refrigerator. On the same day, samples were transported to the laboratory, maintaining assigned storage temperature conditions of each sample until analysis 24 hours after collection. A total of 30 urine samples of 25ml were evaluated.

**Fig 2:**
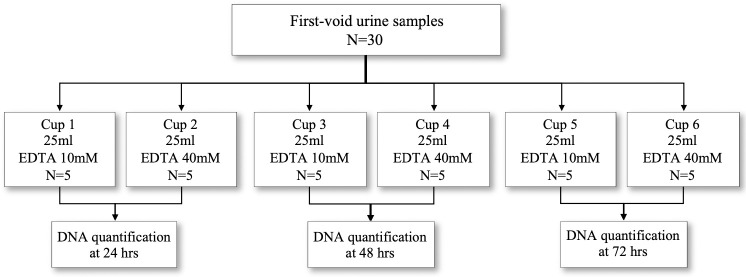
Overview of urine sample collection and processing of second evaluation. Separating samples into two groups with different EDTA concentrations, further divided to examine different lengths of time from urine specimen collection to time of specimen processing. This evaluation was performed as a set of urine samples from five volunteer women, who collected a first-morning, first-void urine sample, in six different cups (25ml each). Three cups contained 10mM EDTA while the other three cups contained 40mM. Samples were then processed at 24, 48 and 72 hours as shown.

A second evaluation working with the same volunteers focused on the best time between urine collection and DNA extraction, in an attempt to avoid the precipitation of protein crystals, microorganism proliferation and DNA degradation. Participants were asked to collect a first-morning, first-void urine sample of 25ml each in six different cups of 25ml each, numbered from one to six. Three cups contained 10mM EDTA while the other half contained 40mM. Samples were then processed at 24, 48 and 72 hours (Fig 2).

**Fig 3:**
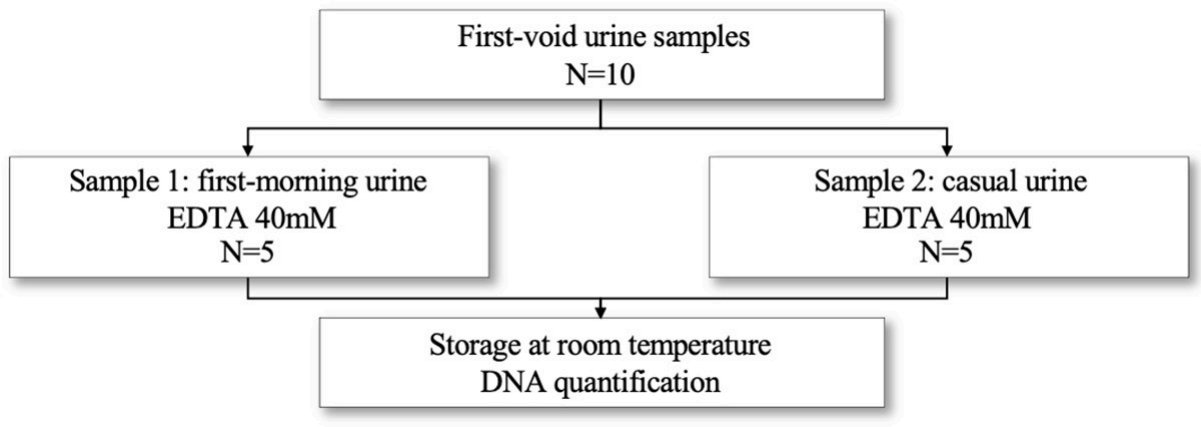
Overview of sample collection and processing for the third evaluation. Two samples were collected from each of the five participants, one from a first-morning micturition and a second one at a convenient time of the day which differed between women and was not prespecified. Samples were then stored at room temperature until DNA extraction 24 hours after collection.

We also determined if a convenience urine sample provided similar or different DNA concentration compared to a first-morning micturition. Two samples were collected from each of the five participants, one from a first-morning micturition and a second one at a convenient time of the day. Samples were then stored at room temperature until DNA extraction 24 hours after collection (Fig 3).

### DNA extraction and quantification process

DNA extraction and quantification in all evaluations of the first phase of this work were performed with a similar procedure. All samples were centrifuged at 3000 rpm for 10 minutes, the pellet was resuspended in 1ml of supernatant and the rest of supernatant was discarded. For extraction, the Gentra Puregene Cell Kit’s (©Qiagen GmbH, Germany) [17] protocol specified by the manufacturer was followed. Then, 5.5ml of lysis solution and 30μl proteinase-K (20mg/ml) were added to the resuspended pellet and later mixed by vortexing for 1 minute. The mix was then incubated at 55°C overnight (15 hours), subsequently mixed again for 1 minute and 2ml of protein precipitation solution added and mixed vigorously for 1 minute. Subsequently, the DNA was quantified by UV absorption using a spectrophotometer (NanoDrop^TM^, ThermoFisherScientific) per the manufacturer’s protocol [18].

### Second phase

We evaluated the agreement performance of our urine sampling storage and processing method among participants of the FASTER Study; using urine samples collected by 95 subjects who were randomly selected among women attending routine cervical cancer screening in a primary health-care clinic in Tlalpan Health Jurisdiction, Mexico City [16]. At the time of appointment for cervical cancer screening, immediately preceding the clinician-collected cervical sample, all women were instructed to collect a urine sample in the restroom of the health-care center. The urine samples were stored according to the optimal method derived from the first phase of the study. These paired samples, standard clinician-collected cervical samples and urine samples- were processed using an automated PCR platform (BD Viper^TM^ System) [19], which has an integrated module for protein denaturation, DNA extraction and HPV genotyping.

Qualitative data assessing the beliefs and attitudes of patients and health care personnel was collected from July to August 2018 to obtain information about current cervical cancer screening and self-collected urine. A group of three interviewers carried out interviews in different health-care centers of the Tlalpan Health Jurisdiction, Mexico City, ensuring participants’ privacy and comfort. Additionally, semi-structured interviews were performed with14 participants of the FASTER study (not included in the aforementioned 95 subjects) to evaluate the acceptability and feasibility as a potential implementation of urine self-sampling in routine cervical cancer screening conditions.

In addition, nine health-care professionals of primary health-care centers and nine decision-makers who witnessed the use of the urine self-sample alternative among participants of the FASTER Study were also interviewed. Interviews with consenting participants took place in private rooms at the health-care centers. Interview guides focused on attitudes, acceptability, and feasibility of urine sampling for primary hrHPV-testing in cervical cancer screening. Field-notes were taken during interviews using open-ended questioning. All interviews were audio-recorded. Finally, we carried out an exploratory micro-cost analysis including the supplies necessary for obtaining a sample at retail pricing.

### Data analysis

For each evaluation in phase I, descriptive analyses were performed to summarize DNA concentration values obtained from different storage conditions. The Wilcoxon test for non-parametric statistics was performed in paired samples to compare DNA median concentrations according to different EDTA concentrations (40mM, 10mM and no EDTA), storage temperatures (4°C and 25°C), duration between sample collection and extraction (24h, 28h, 72h) and different sample collection times (first-morning micturition and convenience sample).

For phase II, we performed descriptive analyses for specific HPV genotype detection and examined correlations between HPV types detected in cervical and urine samples using Cohen’s Kappa coefficient. Stata Corp LLC Stata® Version 14.2 was used for these evaluations.

For the qualitative analysis, we identified emerging themes related to acceptance (ease and comfort) and feasibility perception; Data interpretation was performed by grouping relevant information by category, following standard approaches [20].

## Results

### Optimal EDTA concentration and storage temperature

The mean concentration of DNA obtained from samples stored either at room temperature (≈25°C) or 4°C for a maximum of 72 hours was determined. Specimens stored at ≈25°C without EDTA, 10 mM EDTA or 40 mM EDTA yielded mean concentrations of 30.4, 15.8 and 41.1 ng/μl respectively. The highest EDTA concentration (40mM) offered significantly higher DNA concentrations compared to 10mM EDTA (p = 0.043) and without EDTA (p = 0.06).

Similarly, specimens stored at 4°C, without EDTA, 10mM EDTA or 40mM EDTA yielded mean concentrations of 8.6, 6.0, and 13.0 ng/μl, respectively. Again, a high concentration of EDTA yielded better DNA concentrations against 10mM EDTA (p = 0.043) and without EDTA (p = 0.043).

**Fig 4:**
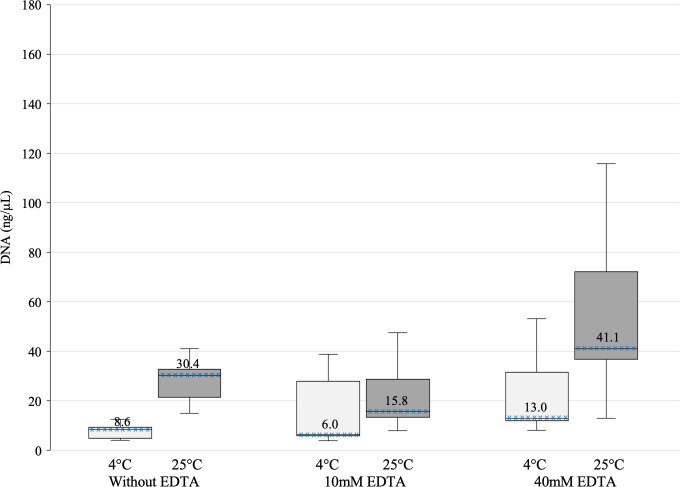
DNA concentration in samples stored in different EDTA concentrations and temperatures. The top of the box represents the upper quartile (p75), the bottom the lower quartile (p25), and the x-patterned line the median (p50), the median is identified above the line. The upper and lower whiskers extend to 1.5 times the interquartile range. The best DNA concentrations resulted from EDTA at 40mM, both at 25°C and 4°C. The best storage temperature was 25°C compared to 4°C, regardless of EDTA concentration. DNA extraction and quantification presented for this particular experiment were performed 24 hours after collection.

The best storage temperature was 25°C compared to 4°C, regardless of EDTA concentration: 30.4 vs. 8.6 ng/μl without EDTA; 15.8 vs. 6.0 ng/μl with 10mM EDTA; and 41.1 vs. 13.0 ng/μl with 40mM EDTA (p = 0.041, p = 0.043, p = 0.043, respectively) (Fig 4). Regarding DNA purity as determined by the 260/280 absorbance (nanometers;nm) ratio for samples preserved at 25°C (median = 1.82, p25 = 1.81, p75 = 1.89) was better than in samples stored at 4°C (median = 1.47, p25 = 0.95, p75 = 1.75). A 260/280 ratio of ~1.8 is generally accepted as “pure” for DNA. On the other hand, a 260/280 ratio <1.6 indicates probable contamination from residual phenol, guanidine, proteins or other reagent used in the extraction protocol [21]. Addicionaly the refrigerated urine samples also showed macroscopic crystalline forms which were absent in samples stored at room temperature.

### Effect of time from collection to processing

**Fig 5:**
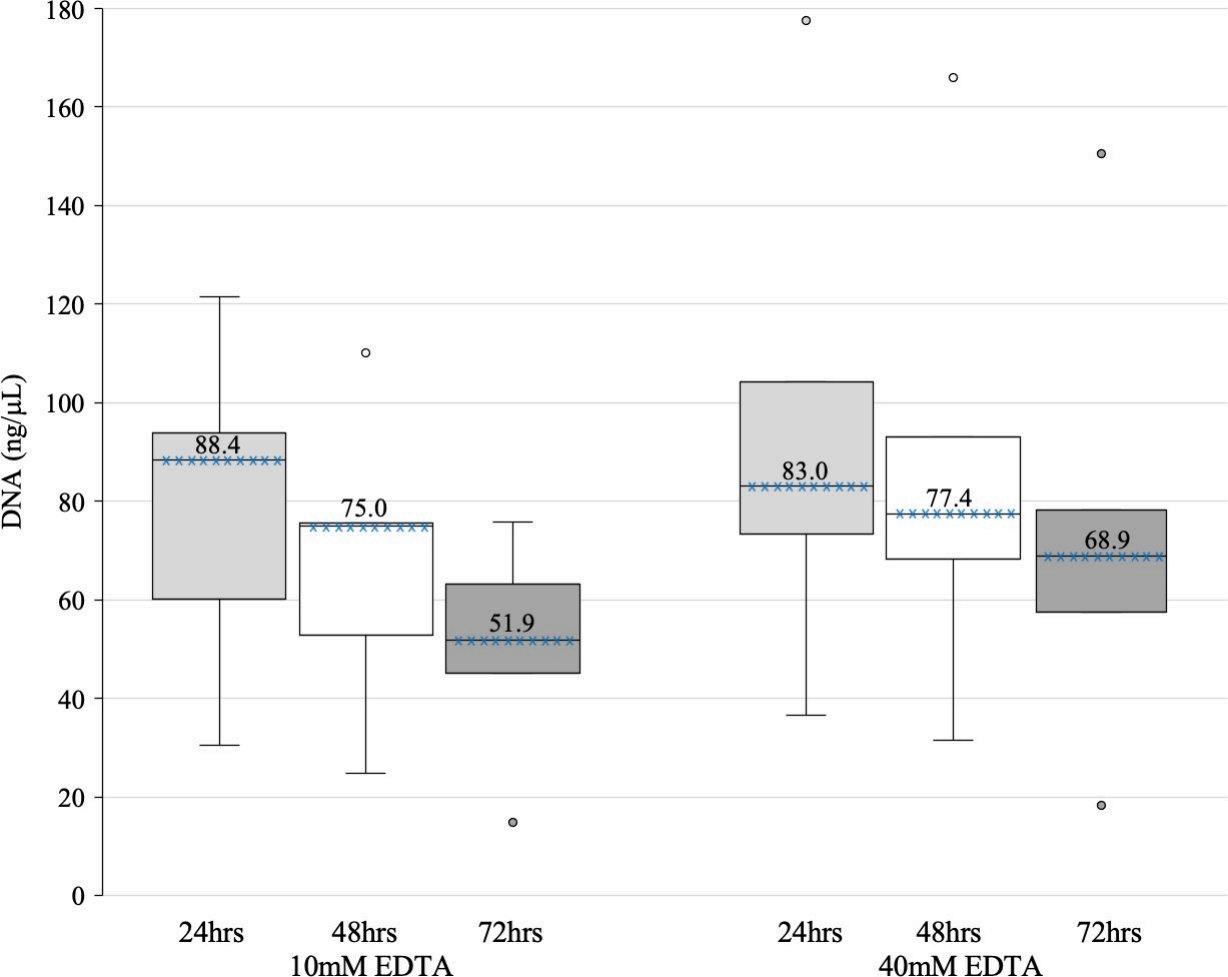
DNA concentration of samples processed at different times after sample collection. The top of the box represents the upper quartile (p75), the bottom the lower quartile (p25), and the line the median (p50), the median is displayed above the line. The upper and lower whiskers extend to 1.5 times the interquartile range, while the dots mark the largest and smallest values of the distribution.

We observed that the time elapsed between sample collection and processing influenced a lower DNA concentration. The median DNA concentrations of samples stored in 10mM EDTA processed at 24, 48 and 72 hours were 88.4, 75.0 and 51.9 ng/μl, respectively. The median DNA concentrations of samples stored in 40mM EDTA were 83.0, 77.4 and 68.9 ng/μl for samples processed at 24, 48 and 72 hours, respectively (Fig 5).

### First-morning urine self-sampling vs. convenience micturition

No significant differences were observed between DNA concentrations from first morning micturition samples [median = 79.5 ng/μl, (p25 = 65.3, p75 = 91.2)] compared to a micturition collected at any time of the day [median = 60.4 ng/μl, (p25 = 53.3, p75 = 67.1)] (p = 0.11 for Wilcoxon test) when stored at room temperature with 40mM EDTA.

### Correspondence of specific hrHPV detection in urine samples vs. cervical samples

We evaluated the agreement of specific hrHPV detection on 95 paired urine samples compared to clinician-collected cervical samples. We used urine samples preserved with the optimal collection method based on the results of our initial studies to achieve optimization. The general characteristics of the participants in this paired comparison are presented in S1 Table of the Supporting information file. The mean age of women was 41.5±9.9 years, 43.2% were 20 to 39 years old, 35.8% were 40 to 49 years old and 21.1% were 50 years old or more. The majority were married or cohabitating (74.7%), 40% reported having had 1 sexual partner in their lifetime whereas 30.5% had had between 2 and 3 partners.

Among the 95 paired samples, all urine and cervical samples had the amplification control target of the β-globin gene detected. In cervical samples, the prevalence of hrHPV was 13.7%, of HPV16 1.1% and 0% for HPV18. Urine samples had a prevalence of hrHPV of 22.1%, 3.2% for HPV16, and 0% positive samples for HPV18.

We observed that all 13 hrHPV positives identified by the HPV testing in cervical samples were successfully detected in urine samples. The specific hrHPV types detected in cervical samples, were also detected in urine samples. In addition, we detected a case with HPV 45 and another with HPV 52 in urine that were not detected in cervical samples. Among these paired samples we observed an acceptable agreement of hrHPV detection in paired urine and cervical samples as shown by a Kappa value of >0.6 (Table 1).

**Table 1:** Type specific HPV positivity differences between urine and cervical samples.

	Cevical sample n (%)	Urine sample n (%)	Kappa values
**hrHPV+**	13 (13.7)	21 (22.1)	0.72
HPV16+	1 (1.1)	3 (3.2)	0.49
HPV18+	0	0	-
HPV45+	0	1 (1.1)	0.00
HPV31+	3 (3.2)	4 (4.2)	0.85
HPV51+	1 (1.1)	3 (3.2)	0.49
HPV52+	0	1 (1.1)	0.00
Pool 1^a^	2 (2.1)	3 (3.2)	0.79
Pool 2^b^	6 (6.3)	8 (8.4)	0.84
Pool 3^c^	2 (2.1)	5 (5.3)	0.56

^a^ Pool 1: HPV33/58

^b^ Pool 2: HPV56/59/66

^c^ Pool 3: 35/39/68.

### Acceptability, feasibility and costs of urine self-sampling

#### Fourteen women were subjected to interview

The majority (64%) had completed basic or middle-level education; half of them lived with a partner and had at least one child. All women stated that urine self-sampling was a more comfortable and acceptable method of cervical cancer screening compared to clinician-directed cervical sampling. They felt that collecting a urine sample was an easy and common procedure, and women were familiar with routine urinalysis. Eliminating the shame associated with clinician-directed cervical sampling, with the added advantages of a substantially faster and more comfortable process along with the possibility of collecting urine at home made the future use of urine samples for the detection of cervical cancer a very attractive alternative.

We also interviewed five physicians, three nurses and one social worker, ages 34 to 62, of which 89% were women. All interviewed agreed that urine collection was a more straightforward strategy for cervical cancer screening than sampling collection requiring a pelvic examination. Furthermore, they felt that urine analysis was an excellent opportunity for women to be able to obtain the sample in their own homes and deliver it to their healthcare centers. Moreover, 78% of physicians considered that urine collection may be implemented as a cervical cancer screening strategy given the convenience and simplicity of the procedure.

Of nine policy decision-makers interviewed, 40% were women, 44% had specialty or postgraduate studies, with several years of experience in the field of preventive medicine. Six of them were heads of departments (i.e., nursing, preventive medicine, cancer screening, epidemiology). All considered urine self-sampling as an alternative that could overcome barriers such as the discomfort associated with pelvic examination and cervical sampling, with a good possibility for its implementation in healthcare centers at no additional cost.

The preliminary cost of a urine sample was estimated at USD $1.10, the details of prices are listed in Table 2.

**Table 2:** Supplies for urine self-sampling.

Supplies	Unit costs*
Urine collection cup (100ml)	$0.35
EDTA Buffer	$0.02
Sterile Conic Tube (50ml)	$0.38
Cell lysis	$0.14
Semiautomatic micropipette and tips	$0.05
Sterile Nitrile gloves	$0.01
Cryovials 2mL (Sterile)	$0.15
Packaging and storage	$0.03
**Total cost per one urine sample**	**$1.13**

*Costs are expressed in USD, exchange rate as of June 2020.

## Discussion

Our results suggest that DNA for HPV-testing can be obtained from urine samples with only a simple solution of 40mM EDTA and can be stored at room temperature up to 72 hours before processing. Urine HPV-testing, employing this low-cost sampling procedure, offers good agreement with paired clinician-collected cervical samples. Furthermore, urine self-sampling seems to be highly acceptable and is an affordable method with good potential for implementation within the cervical cancer screening program in Mexico.

Vorsters et al. previously tried a new in-house buffer for urine samples destined for HPV detection that contained bovine serum albumin, a microbicide and a fungicide in addition to the chelating agent [7]. Although this might be a suitable buffer, it has been previously reported that only adding EDTA as a chelating agent may be sufficient for good DNA preservation [5,7,10]. Our findings are consistent with these reports as we were able to obtain optimal results using only 40mM EDTA, which can be expected to keep costs low.

Room temperature was not suitable for storing urine samples in the absence of EDTA. In contrast, studies by Bosschieter et al., Cannas et al. and Augustus et al. found that appropriate DNA concentrations could be extracted from urine stored at room temperature for several days as long as EDTA was added [9,10,22]. In our study, the DNA quantification from urine samples stored at room temperature (25°C) was superior to refrigeration at 4°C (p = 0.004). These findings are similar to those of Augustus et al., who found that, when using preservatives, urine samples stored at room temperature and at 30°C showed higher concentrations of DNA and higher stability over time when compared to samples stored at 4°C [22]. Furthermore, as demonstrated by a higher absorbance ratio 260/280, samples stored at room temperature had a higher purity. These findings in refrigerated samples could be associated with the presence of previously mentioned crystalline formations. A previous study also reported that crystalline formations occurred when refrigerating urine and after microscopic examination identified them as urate and phosphate crystals [23]. They linked the presence of these crystals in urine to lower DNA concentrations and lower 260/280 ratios, suggesting that these crystals interfered with DNA purification. Further studies could help establish how different temperatures affect DNA concentrations, as there are geographical regions in which room temperature will likely exceed 30°C. More studies could also help determine the role of crystals in DNA purification from urine samples.

Regarding time from collection to processing, we found that processing within the first 72 hours yielded DNA concentrations appropriate for HPV testing. These results are consistent with previous findings documenting that DNA concentration decreases over time [5,9]. We did not find any statistically significant difference when comparing first-morning urine against a convenience sample, which confirms findings from previous authors [11,12].

The cost estimate of a urine sample in our study ~ USD $1.10, was much less than currently available commercial kits [24,25]. Without considering the cost of handling and processing samples for HPV screening, one cervical sample vial costs USD $2.42, plus the additional cost of collecting the cervical sample (approximately USD $2.00) [26]. We are not aware of studies estimating the cost of a urine test and comparing it to a standard detection test. Nevertheless, our findings show a lower cost for obtaining a urine sample versus a clinician-collected cervical sample, which offers an additional advantage of this alternative, making it more attractive and promising. Based on our results, a simple and affordable collection and storage procedure is possible and can be proposed for potential use within the cervical cancer screening program.

hrHPV detection in urine samples has previously been reported with acceptable agreement compared to clinician-directed cervical samples [12–14,23,27]. However, as previously documented by Van Keer et al. [28], urine samples detected a larger proportion of HPV infections than cervical samples. This difference could be due to the fact that urine contains sloughed cells and mucus from many parts of the genitourinary tract that contaminate the first-void urine fraction, whereas cervical samples are largely cervical cells. Thus, urine self-samples could generate an increase in false positives; triage alternatives should be used to improve screening effectiveness.

We defined the degree of agreement according to the guidelines outlined by Landis and Koch in 1977: < 0 no agreement, 0.01 to 0.2 slight agreement, 0.21 to 0.40 fair agreement, 0.41 to 0.60 moderate agreement, 0.61 to 0.80 substantial agreement and 0.8 to 1.0 almost perfect agreement [29]. In our study the overall Kappa value for HPV was 0.72, with a Kappa for HPV16 of 0.49, indicating at least a moderate agreement. The agreement between urine and HPV samples for HPV detection is consistent with previously reported Kappa values, which vary from 0.35 up to 0.96 [12,14,27,28]. All HPV infections observed in cervical samples were detected in urine.

Our qualitative analysis reveals that women consider acceptable urine self-sampling and preferred this because of characteristics such as comfort, simplicity and speed of collection. Our participants consider it could be a feasible strategy for implementation at their healthcare centers. These findings are similar to those reported in previous studies, highlighting the advantages of urine collection as a method for HPV DNA detection [13,30–32]. Sellors et al. in Canada, reported acceptability of 98.4% for urine sample collection compared to 79% for cervical samples [33]. A study by Sy et al. done on Micronesian women described urine sampling as more comfortable compared to cervical sampling, by 95% and 82%, respectively [32], while another study reported that urine collection reduced fear and anxiety in participants, proposing it as an attractive alternative that could become a routine procedure [30]. Most healthcare professionals we interviewed (78%) were involved in cancer screening programs and they considered it feasible to implement urine collection as a good alternative for HPV-testing. To our knowledge, this evaluation among healthcare professionals and personnel responsible for cervical cancer screening program has not been previously reported.

In accordance with earlier reports, we believe that the urine self-sampling method has the potential to impact screening coverage [31,34]. In high-income regions such as France, a study observed that urine samples for HPV-testing could become a relevant method for providing screening to underserved women in rural areas and to women who refuse the more invasive sampling required for cervical cytology testing. This would lead to an increase in coverage and timely treatment of high-grade lesions, potentially diminishing mortality rates and associated treatment costs of cervical cancer [34].

One of the strengths of our study was our focus on real-world applicability. Phase one focused on pre-analytical conditions geared towards techniques most likely to be applied to our environment with favorable results, such as room temperature storage, processing within 72 hours, and use of a simple EDTA-based solution for preservation. Furthermore, the second phase, albeit with a small number of participants, was executed applying the lessons learned during phase one, with valuable input on acceptability and feasibility from both patients and healthcare professionals actively involved in cervical cancer early detection programs in Mexico.

However, as mentioned above, an important limitation of this study was the small sample size of the second phase which was descriptive to support future appropriately powered investigations. Our main objective was to provide early evidence about whether urine sample using a new low-cost method could be successful in subsequent diagnostic trials.

Nevertheless, acknowledging that the second phase of our study was to evaluate the agreement between hrHPV-testing in urine samples and regular cervical samples, we performed a post-hoc power calculation to detect differences in HPV detection for paired samples. All HPV positives cases identifying in cervical samples were successfully detected in urine samples with a power of 81.79%, with an alpha of 0.05 using paired samples from 95 participants.

This study showed only the concordance between urine and cervical sample for HPV detection. For HPV tests using self-collected urine to be clinically applied, further investigation will be required to measure the clinical performance of HPV tests with evaluation of CIN2+ detection.

Overall, we found urine-self sampling to be less expensive than cervical samples, was able to overcome technical and cultural barriers, and had an acceptable agreement with clinician-collected cervical sampling in the detection of HPV. Our findings and those of previous studies, support proposing urine as promising alternative for increasing coverage of cervical cancer screening programs worth further evaluation. Our findings also suggest that a urine sampling strategy is worth further evaluation as it is also seen as feasible by healthcare professionals and accepted by patients for implementation as part of the cervical cancer screening program in Mexico.

## Supporting information

S1 File Interview guides. Interview guides applied for women, healthcare professionals and decision-makers. The Interview guides were focused on attitudes, acceptability, and feasibility of urine sampling for primary hrHPV-testing in cervical cancer screening.
(PDF)

S2 File Dataset. DNA quantification and HPV results.
(XLSX)

S1 Table General characteristics of the participants. Demographic and clinical characteristics of the 95 participants in the study.
(PDF)
