# Supplementary material for: Performance of an affordable urine self-sampling method for human papillomavirus detection in Mexican women
Source: PLoS One. 2021 Jul 21;16(7):e0254946. doi: 10.1371/journal.pone.0254946 (PMC8294492; doi:10.1371/journal.pone.0254946)
Supplement: S1 File — Interview guides applied for women, healthcare professionals and decision-makers. The Interview guides were focused on attitudes, acceptability, and feasibility of urine sampling for primary hrHPV-testing in cervical cancer screening. (PDF) [file pone.0254946.s001.pdf]

Instituto Nacional de Salud Pública

Guía de entrevista para mujeres

Folio de identificación

Esta guía de entrevista aplica para mujeres entre 25 y 45 años, participantes del proyecto FASTER. Recuerde grabar la respuesta de aceptación (consentimiento verbal).

Percepciones sobre la colección de orina para la detección oportuna de cáncer cervical

1. Cómo parte del estudio FASTER le pedimos nos regalara un poco de orina para evaluar si es posible identificar el virus de papiloma humano y detectar oportunamente el cáncer cervical. ¿Qué pensó usted cuando le solicitaron colectar orina? ¿Qué ideas le vinieron a la cabeza?

2. Me podría platicar cómo fue su experiencia ¿fue fácil o difícil seguir las instrucciones?

3. Si en el futuro pudiéramos hacer las pruebas para la detección de cáncer cervical a través de la orina ¿qué opinaría? ¿usted aceptaría?

4. ¿Qué necesitamos decirles a las mujeres para que acepten la colección de orina como medio de detección de cáncer cervical? ¿Qué cree que opinarían?

5. En su opinión ¿sería posible llevar a cabo esta propuesta en este centro de salud? ¿qué necesitamos? ¿qué nos hace falta?

Agradezca el aceptar la entrevista y el tiempo para llevarla a cabo.

Instituto Nacional de Salud Pública

Women's Interview Guide

Identification number

This interview guide applies to women between the age of 25 and 45, FASTER project participants. Remember to record the acceptance response (verbal consent).

Perceptions on Urine Collection for Early Cervical Cancer Detection

1. As part of the FASTER study we asked you to give us some urine to assess whether it is possible to identify human papillomavirus and detect cervical cancer in a timely manner. What did you think when you were asked to collect urine? What ideas came to mind?

2. Could you tell me how your experience went, was it easy or difficult to follow the instructions?

3. If in the future we could do cervical cancer screening through urine, what would you think? Would you accept?

4. What do we need to tell women to accept urine collection as a means of cervical cancer screening? What do you believe they would think?

5. In your opinion, would it be possible to carry out this proposal in this health center? What is needed? What are we missing?

Thank the participants for their time and input.

Instituto Nacional de Salud Pública

Guía de entrevista para el personal de salud y tomadores de decisiones

Folio de identificación

Esta guía de entrevista aplica a personal de salud (médicos(as)/enfermeras(os) así como tomadores de decisión/directivos. Recuerde grabar la respuesta de aceptación (consentimiento verbal).

Percepciones sobre la colección de orina para la detección oportuna de cáncer cervical

1. ¿Qué opina sobre colección de orina para la detección oportuna de cáncer cervical?

2. ¿Qué tan factible sería implementarla es este centro de salud? ¿por qué?

3. ¿Qué dificultades y facilitadores habría?

4. ¿Qué recomendarías para implementarla?

5. ¿Cree que sus compañeros aceptarían esta propuesta? ¿Por qué?

6. ¿Cree que las mujeres acepten esta propuesta? ¿Por qué?

Agradezca el aceptar la entrevista y el tiempo para llevarla a cabo.

Instituto Nacional de Salud Pública

Health personnel´s Interview Guide and decision makers

Identification number

This interview guide applies to health personnel (doctors/nurses) as well as decision makers/management. Remember to record the acceptance response (verbal consent).

Perceptions on Urine Collection for Early Cervical Cancer Detection

1. What do you think about urine collection for timely cervical cancer screening?

2. How feasible would it be to implement it in this health center? Why?

3. What obstacles and advantages would there be?

4. What would you recommend for its implementation?

5. Do you think your colleagues would accept this proposal? Why?

6. Do you think women would accept this proposal? Why?

Thank the medical personnel for their participation and input.
