# Supplementary material for: Performance of an affordable urine self-sampling method for human papillomavirus detection in Mexican women
Source: PLoS One. 2021 Jul 21;16(7):e0254946. doi: 10.1371/journal.pone.0254946 (PMC8294492; doi:10.1371/journal.pone.0254946)
Supplement: S1 Table — Demographic and clinical characteristics of the 95 participants in the study. (PDF) [file pone.0254946.s003.pdf]

Table 3. General characteristics of the participants (n=95)

Demographic and clinical characteristics	n	(%)
**Age group (years), mean (sd)**	41.54	(9.87)
25 to 29	15	(15.79)
30 to 39	26	(27.37)
40 to 49	34	(35.79)
50 to 59	16	(16.84)
60 or more	4	(4.21)
**Marital status**		
Married/Cohabitating	71	(74.74)
Divorced/separated/widowed	6	(6.32)
Single	18	(18.95)
**Lifetime sexual partners**		
1	38	(40.00)
2 to 3	41	(43.16)
4 or more	16	(16.84)
**Parity**		
0	25	(26.32)
1 to 3	57	(60.00)
4 or more	13	(13.68)
